# Microdialysis Determination of Cefquinome Pharmacokinetics in Murine Thigh From Healthy, Neutropenic, and *Actinobacillus pleuropneumoniae-*Infected Mice

**DOI:** 10.3389/fphar.2019.00249

**Published:** 2019-03-12

**Authors:** Longfei Zhang, Lihua Yao, Zheng Kang, Zilong Huang, Xiaoyan Gu, Xiangguang Shen, Huanzhong Ding

**Affiliations:** Guangdong Key Laboratory for Veterinary Drug Development and Safety Evaluation, South China Agricultural University, Guangzhou, China

**Keywords:** cefquinome, microdialysis, pharmacokinetics, *in vivo*, dialysis

## Abstract

This study was aimed at applying microdialysis to explore cefquinome pharmacokinetics in thigh and plasma of healthy, neutropenic, and *Actinobacillus pleuropneumoniae*-infected mice. The relative recoveries (RRs) were tested *in vitro* by dialysis and retrodialysis and *in vivo* by retrodialysis. ICR mice were randomly divided into four groups: H-40 (healthy mice receiving cefquinome at 40 mg/kg), H-160, N-40 (neutropenic mice), and I-40 mg/kg (thigh infected-mice with *A. pleuropneumoniae*). After cefquinome administration, plasma was collected by retro-orbital puncture and thigh dialysate was collected by using a microdialysis probe with Ringer’s solution at a perfusion rate of 1.5 μL/min. Plasma and thigh dialysate samples were assessed by HPLC–MS/MS and analyzed by a non-compartment model. The mean *in vivo* recoveries in the thigh were 39.35, 38.59, and 37.29% for healthy, neutropenic, and infected mice, respectively. The mean plasma protein-binding level was 16.40% and was independent of drug concentrations. For all groups, the mean values of the free AUC_inf_ in plasma were higher than those in murine thigh, while the elimination *T*_1/2β_ for plasma were lower than those for murine thigh. Cefquinome penetration (AUC_thigh_/AUC_plasma_) from the plasma to thigh was 0.76, 0.88, 0.47, and 0.98 for H-40, N-40, I-40, and H-160 mg/kg, respectively. These results indicated that infection significantly affected cefquinome pharmacokinetics in murine thigh. In conclusion, we successfully applied a microdialysis method to evaluate the pharmacokinetics of cefquinome in murine thigh of healthy, neutropenic, and *A. pleuropneumonia*-infected mice and the pharmacokinetics of cefquinome was obviously affected by infection in thigh.

## Introduction

Cefquinome is a fourth-generation broad-spectrum cephalosporin that has been solely used in veterinary therapy. It was approved to treat respiratory tract disease, acute mastitis, and foot rot in cattle; calf septicemia; metritis–mastitis–agalactia syndrome in sows; and respiratory diseases in pigs (Nahler, 2009). The pharmacokinetics of cefquinome in plasma have been studied in various species, such as mice (Shan et al., 2014; Wang et al., 2014), rabbits (Hwang et al., 2011; Xiong et al., 2016), and pigs (Zhang et al., 2014), and the results have been used to design dose regimens associated with the pharmacodynamics in target tissues. However, it is widely recognized that systemic plasma concentrations may not adequately predict tissue drug concentrations. Therefore, it is necessary to monitor unbound concentrations of antibacterial drugs at the target site. Microdialysis, a sampling technique that can be used to monitor unbound drug concentrations in the interstitium of any virtual tissue over time, can adequately address this need.

Microdialysis was first applied in the early 1960s, when animal tissue biochemistry was studied by inserting push–pull cannulas and dialytrodes, among others, into the tissue of interest, most often the cerebral tissue of rodents (Chaurasia et al., 2007). Microdialysis is a minimally invasive technique that allows continuous tissue sampling of free drug concentrations in the tissue of interest (Brunner et al., 2005). The pharmacokinetics of antibiotics have been recently studied in some organs such as the lung. One group (Yang et al., 2017) used microdialysis to evaluate the pharmacokinetics of florfenicol in the lung after a single intramuscular administration. Another group (Bernardi et al., 2017) applied microdialysis to evaluate the lung penetration of tobramycin in rats. Therefore, microdialysis is a valuable and efficient method to study antibacterial drugs in human and animals.

In the present study, the microdialysis technique was first applied to determine the pharmacokinetics of cefquinome in a murine thigh model. The aim of our experiment was to elucidate the concentration-versus-time profile of cefquinome in the thigh of mice by using the microdialysis technique, and another objective was to evaluate the differences in cefquinome pharmacokinetics in the plasma and thigh tissues in healthy, neutropenic, and infected mice by *Actinobacillus pleuropneumoniae* CVCC 259 in the thigh.

## Materials and Methods

### Bacteria, Media, and Antibiotic

The *A. pleuropneumoniae* standard strain, CVCC259, was purchased from the China Veterinary Culture Collection Center (Qingdao, China). Cefquinome standard was provided by China Institute of Veterinary Drugs Control (Beijing, China). Pentobarbital sodium was purchased from Jian Yang Biotechnology Co., Ltd. Tryptic soy broth (TSB) and MH agar (MHA) were purchased from Guangdong Huankai Microbial Technology. Nicotinamide adenine dinucleotide (NAD, lot: 20160810) and heparin sodium salt (lot: 20170906) were purchased from MYM Biological Technology Company Limited. Newborn bovine serum was provided by Guangzhou Ruite Biotechnology Ltd. Ringer’s solution and cyclophosphamide were purchased from Shanghai Yuanye Bio-Technology Co., Ltd.

### Animals and Housing

Six-week-old, specific-pathogen-free female ICR mice (weight, 25–30 g) were provided by Hunan SJA Laboratory Animal Co., Ltd. (Hunan, China). All the animals were housed in the Laboratory Animal Center of South China Agricultural University. Water and fodder were provided *ad libitum*. They were acclimatized for 7 days prior to the experiment. All the experimental protocols were approved by the Committee on the Ethics of Animals of South China Agricultural University (Approval number: 2017029).

### Neutropenic Mouse and Murine Thigh Infection Model

Neutropenia was induced by cyclophosphamide administration at 4 (150 mg/kg) and 1 day (100 mg/kg) prior to infection by intraperitoneal injection. *A. pleuropneumoniae* was incubated in TSB and MHA supplemented with 4% newborn bovine serum and 1% 1 mg/mL NAD. The logarithmic-phase bacteria were used after incubation for 8 h in TSB in a 200-rpm/min humidified incubator (37°C, 5% CO_2_). After tenfold dilution, inocula with a final bacterial concentration ranging from 10^6^ to 10^7^ CFU/mL (0.1 mL/thigh) were injected into each thigh of the mice by intramuscular injection. Two hours after infection, the murine thigh infection model was established.

### Microdialysis and Calibration

The microdialysis system included a BASi Syringe Pump which consist of a single syringe pump drive, a drive controller (Worker Controller), and a gas-tight syringe (1.0 mL) connected to an MD-2000 linear microdialysis probe (membrane length: 10 mm, cutoff: 30 kDa). All microdialysis devices applied in our experiment were provided by Bioanalytical System, Inc. (West Lafayette, IN, United States).

For calibrating the probe, the relative recoveries (RRs) were tested by dialysis and retrodialysis *in vitro* according to a previous report (Araujo et al., 2008) and calculated using formulas (1) and (2), respectively.

(1) $${\text{RR}}_{\text{dialysis}}(\%)\;\text{=}\;(C_{\text{dial}}\text{/}C_{\text{ext}})\;\times \;\text{100}$$

(2) $${\text{RR}}_{\text{retrodialysis}}(\%)\;\text{=}\;((C_{\text{perf}}–C_{\text{dial}})\text{/}C_{\text{perf}})\;\times \;\text{100}$$

where *C*_dial_ is the concentration of cefquinome in the dialysate, *C*_ext_ is the concentration of cefquinome in the Ringer’s solution surrounding the microdialysis probe, and *C*_perf_ is the concentration of cefquinome in the perfusate. Different cefquinome concentrations (50, 100, and 500 ng/mL) in Ringer’s solution and various perfusion flow rates (0.5, 1.0, 1.5, and 2.0 μL/min) were examined. Each experiment was repeated three times.

For *in vivo* calibration, the RRs for the thigh of healthy, neutropenic, and infected mice were determined by retrodialysis using Ringer’s solution with cefquinome (500 ng/mL) at a perfusion flow rate of 1.5 μL/min according to a previously reported protocol with slight modification (Bernardi et al., 2017). The thigh dialysate was collected at 15-min intervals over 6 h. Each experiment was repeated three times.

### Determination of Plasma Protein Binding

Plasma protein binding in healthy, neutropenic, and infected mice was also determined by microdialysis *in vitro*. The RR in plasma was detected by retrodialysis. In detail, after the probe was immersed into 1 mL of blank plasma, Ringer’s solution containing cefquinome (500 ng/mL) was perfused at a flow rate of 1.5 μL/min. After equilibration for 1 h, four samples were collected at 30-min intervals. The RR was calculated by using formula (2). The free concentration of cefquinome in plasma spiked with cefquinome (5, 50, and 500 ng/mL) was determined by the dialysis method. After detecting the concentration in total plasma and dialysate, the free fraction in plasma was defined as the ratio of the free concentrations and the total concentration.

### Dose Regimens and Sample Collection

The mice were randomly divided into four groups: H-40 and H-160 (healthy mice receiving cefquinome at 40 and 160 mg/kg), N-40 (neutropenic mice receiving cefquinome at 40 mg/kg), and I-40 (neutropenic mice infected with *A. pleuropneumoniae* receiving cefquinome at 40 mg/kg). Cefquinome was administered in a single dose by subcutaneous injection.

For plasma collection, 100 μL blood samples was obtained by a retro-orbital puncture from each of three mice at the following time points: 0.083, 0.17, 0.25, 0.5, 1, 2, 3, and 4 h after administration and each dosage group repeated eight times. The blood samples were collected in 1.5-mL centrifuge tubes with 2% heparin sodium as an anticoagulant. The samples were then centrifuged at 3,000 ×*g* at 4°C for 10 min. The plasma samples were transferred into cryotubes and stored at -20°C until analysis within 2 weeks.

For thigh microdialysis experiments, the microdialysis probe was implanted into the thigh 6 h prior to the experiment. The mice were anesthetized by 1.5% pentobarbital sodium at 0.005 mL/g by intraperitoneal injection. After stable general anesthesia was achieved, an introducer needle (25G needle) was inserted through the thigh muscle. The MD-2000 linear probe was inserted through the introducer needle. The needle was then removed and the microdialysis membrane was kept entirely embedded in the thigh muscle. The inlet and outlet tubes of the probe were pasted to the skin using medical tapes to fix the microdialysis membrane. The probe was then connected to the syringe pump drive and perfused with perfusate (Ringer’s solution) at a flow rate of 1.5 μL/min to stabilize the microdialysis system until the mice were revived consciousness. After administration of cefquinome, the thigh dialysate samples were collected from one mouse (one thigh of each mouse) at the following time points: 0, 0.25, 0.5, 0.75, 1, 2, 3, 4, and 6 h and each dosage group repeated eight times. The perfused rate was 1.5 μL/min, and the sampling interval was 15 min. The dialysates were placed into 0.5-mL centrifuge tubes and stored at -20°C until analysis within 2 weeks. During the experiment, all mice received Ringer’s solution to supply body fluid.

### Determination of Cefquinome Concentration in Plasma and Thigh Dialysate

Cefquinome concentrations in the plasma and thigh dialysate were detected using an Agilent 1200 series HPLC unit and an Agilent 6410 triple quadrupole mass spectrometer equipped with an electrospray ionization source (HPLC–MS/MS, Agilent Technologies). The analysis method was used as described by Zhang et al. (2014) with a little modification.

Briefly, after thawing at room temperature, 20 μL of plasma and 20 μL of acetonitrile were mixed in a 1.5-mL centrifuge tube. After vortexing for 30 s, the mixture was centrifuged at 12,000 ×*g* for 10 min. Next, 20 μL of clear supernatant was extracted and mixed with 80 μL of ultrapure water in a fresh vial. After mixing for 15 s, the samples were filtered through a 0.22-μm nylon syringe filter (Jin Teng Experiment Equipment Company) and transferred into an autosampler vial. For calibration, the standard curve (*R*^2^ > 0.99) was defined by six calibration standards of cefquinome with a final concentration ranging from 5 to 200 ng/mL. The lower limit of quantification (LLOQ) in the plasma was 5 ng/mL.

For the dialysate, 20 μL of sample was added to 80 μL of ultrapure water in a centrifuge tube. After mixing, the mixture was transferred to an autosampler vial. The standard curve (*R*^2^ > 0.99) was determined using eight calibration standards of cefquinome ranging from 2.5 to 200 ng/mL. The LLOQ was 2.5 ng/mL in dialysate.

### Pharmacokinetics Analysis

To calculate the pharmacokinetic parameters, a WinNonlin software (version 5.2, Pharsight Corporation) was used. The pharmacokinetic parameters of free cefquinome in plasma and thigh were both determined using a non-compartment model. The AUC, *C*_max_, mean residence time, and terminal elimination *T*_1/2β_ were estimated. All the data were listed as geometric mean ± SD values and determined by averaging the calculated parameters for the drug in each mouse. One-way ANOVA was used to analyze the difference in cefquinome PK parameters among the healthy, neutropenic, and infected mice. *P* < 0.05 was considered to indicate significant differences. Statistical analyses were performed using Statistical Product and Service Solution (version 22, IBM).

## Results

### RRs of the Microdialysis Probe

**FIGURE 1:**
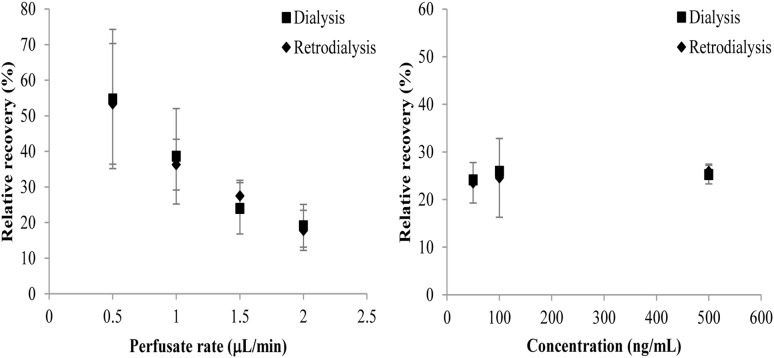
Influence of cefquinome concentrations (right panel) and perfusion fluid flow rates (left panel) on *in vitro* RRs determined by dialysis (■) and retrodialysis (♦). Each symbol represents the mean value ± SD.

The *in vitro* RRs of the microdialysis probe are shown in Figure 1. There was no statistical difference in RRs determined using dialysis or retrodialysis regardless of different flow rates or concentrations. When the flow rate increased from 0.5 to 2 μL/min, the RRs determined by dialysis and retrodialysis decreased from 54.72 to 19.11% and from 53.35 to 17.81%, respectively. The RRs were stable when the concentration of cefquinome increased from 50 to 500 ng/mL. These results indicated that the *in vivo* probe RR could be determined by retrodialysis. Considering the short interval and better RR, the flow rate of 1.5 μL/min (RRs: 24.01 and 27.52% for dialysis and retrodialysis, respectively) was chosen for the following experiments.

**FIGURE 2:**
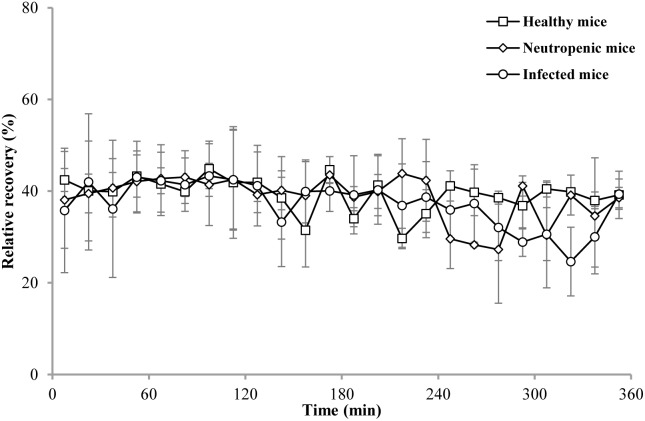
*In vivo* RRs of cefquinome in the thigh of healthy (□), neutropenic (♢), and infected (○) mice. Each symbol represents the mean value ± SD.

The *in vivo* RRs of the probe in the thigh of healthy, neutropenic, and infected mice are shown in Figure 2. All *in vivo* RRs were higher than the *in vitro* RRs. The RRs were stable for 6 h and the mean values were 39.35, 38.59, and 37.29% for healthy, neutropenic, and infected mice, respectively.

### Pharmacokinetics of Free Cefquinome in Plasma and Thigh

With regard to plasma-binding proteins, there was no significant difference when the concentration of cefquinome increased from 5 to 500 ng/mL. The protein binding in the healthy, neutropenic, and infected mice was no difference and the mean value was 16.4%. In this study, the free fraction (0.836) was applied to predict the free concentration of cefquinome in plasma based on the total concentration.

**FIGURE 3:**
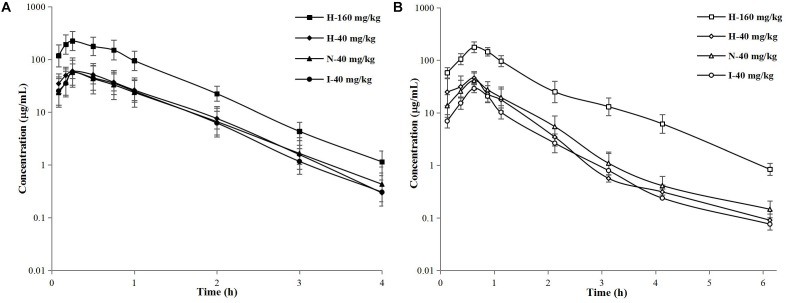
Semilogarithmic concentration–time curves of free cefquinome in murine plasma **(A)** and thigh **(B)** after subcutaneous administration of 40-mg/kg single doses in healthy, neutropenic, and infected mice and 160-mg/kg doses in healthy mice. Solid and open symbols represent the concentrations in murine plasma and thigh. Each symbol represents the mean value ± SD.

The concentration–time curves of free cefquinome in plasma and thigh are shown in Figure 3. After analyzed by a non-compartment model, the pharmacokinetic parameters of free cefquinome in plasma and thigh are listed in Tables 1, 2, respectively. A comparison of the PK parameters of free cefquinome in plasma in different groups revealed that there was almost no difference in *T*_1/2β_ and *C*_max_ at the dosage of 40 mg/kg. However, a lower AUC was observed for neutropenic and infected mice than healthy mice. The *C*_max_ and AUC were linear increased when the dose reached 160 mg/kg in healthy mice.

**Table 1:** Pharmacokinetic parameters of free Cefquinome in murine plasma.

Parameters (unit)	H-40 mg/kg	N-40 mg/kg (P)	I-40 mg/kg (P)	H-160 mg/kg
T_1/2β_ (h)	0.44 ± 0.07	0.48 ± 0.08 (0.366)	0.43 ± 0.09 (0.716)	0.45 ± 0.07
*T*_max_ (h)	0.25	0.25	0.25	0.25
*C*_max_ (μg/mL)	59.78 ± 22.28	56.63 ± 51.21 (0.884)	56.15 ± 41.13 (0.867)	223.13 ± 115.18
AUC_last_ (μg⋅h/mL)	64.47 ± 30.90	56.51 ± 50.89 (0.717)	57.35 ± 31.17 (0.745)	230.64 ± 115.73
AUC_inf_ (μg⋅h/mL)	64.66 ± 31.01	56.84 ± 51.24 (0.723)	57.57 ± 37.31 (0.748)	231.41 ± 116.12
Vd/F (L/kg)	0.64 ± 0.67	1.56 ± 1.29 (0.084)	0.88 ± 0.77 (0.641)	0.56 ± 0.25
Cl/F (L/kg)	0.94 ± 0.77	2.45 ± 2.07 (0.073)	1.52 ± 1.35 (0.470)	0.8 ± 0.38
MRT_0-last_ (h)	0.82 ± 0.07	0.86 ± 0.06 (0.286)	0.83 ± 0.06 (0.676)	0.80 ± 0.05
AUC_inf_/dose (kg⋅h/mL)	1.62 ± 0.83	1.42 ± 1.37 (0.925)	1.44 ± 1.00 (0.748)	1.45 ± 0.78


*T_1/2β_, elimination half-life; T_max_, time of maximum concentration; C_max_, maximum concentration; AUC_last_, area under the plasma concentration–time curve (AUC) computed from time zero to the time of the last positive Y value; AUC_inf_, AUC from time zero extrapolated to infinity; MRT_last_, mean residence time when the drug concentration is based on values up to and including the last measured concentration; Vd/F, volume of distribution during the terminal phase of pseudo-equilibrium scaled by bioavailability; Cl/F, body clearance scaled by bioavailability. All the PK values of cefquinome are listed as mean value ± SD. P-values were acquired by one-way ANOVA statistical analysis after compared to health group. P < 0.05 was considered to indicate significant differences and high values indicate no difference with health group.*

**Table 2:** Pharmacokinetic parameters of free cefquinome in murine thigh.

Parameters (unit)	H-40 mg/kg	N-40 mg/kg (P)	I-40 mg/kg (P)	H-160 mg/kg
*T*_1/2β_ (h)	1.04 ± 0.48	0.87 ± 0.30 (0.424)	1.06 ± 0.36 (0.911)	0.81 ± 0.09
*T*_max_ (h)	0.625	0.625	0.625	0.625
*C*_max_ (μg/mL)	47.22 ± 13.37	45.61 ± 18.92 (0.912)	27.79 ± 5.02 (0.003)	177.65 ± 47.06
AUC_last_ (μg⋅h/mL)	48.84 ± 15.08	49.99 ± 22.38 (0.912)	27.01 ± 3.00 (0.003)	226.90 ± 37.14
AUC_inf_ (μg⋅h/mL)	49.01 ± 15.00	50.15 ± 22.46 (0.913)	27.16 ± 3.01 (0.003)	227.87 ± 37.23
Vd/F (L/kg)	1.60 ± 0.96	1.48 ± 1.01 (0.811)	2.23 ± 0.83 (0.215)	0.85 ± 0.20
Cl/F (L/kg)	1.00 ± 0.39	1.14 ± 0.61 (0.551)	1.45 ± 0.18 (0.066)	0.72 ± 0.11
MRT_0-last_ (h)	0.89 ± 0.12	1.04 ± 0.12 (0.035)	1.01 ± 0.13 (0.087)	1.21 ± 0.17
AUCinf/dose (kg⋅h/mL)	1.23 ± 0.37	1.25 ± 0.56 (0.913)	0.68 ± 0.08 (0.03)	1.42 ± 0.25
AUC_thigh_/AUC_plasma_	0.76	0.88	0.47	0.98


*T_1/2β_, elimination half-life; T_max_, time of maximum concentration; C_max_, maximum concentration; AUC_last_, area under murine thigh concentration–time curve (AUC) computed from time zero to the time of the last positive Y value; AUC_inf_, AUC from time zero extrapolated to infinity; MRT_last_, mean residence time when the drug concentration is based on values up to and including the last measured concentration; Vd/F, volume of distribution during the terminal phase of pseudo-equilibrium scaled by bioavailability; Cl/F, body clearance scaled by bioavailability. All the PK values of cefquinome are listed as mean value ±SD. P-values were acquired by one-way ANOVA statistical analysis after compared to health group. P < 0.05 was considered to indicate significant differences and high values indicate no difference with health group.*

For PK parameters of free cefquinome in thigh, the *C*_max_ and AUC were significantly lower in the infected mice than in the healthy and neutropenic mice. The *C*_max_ and AUC increased in a linear fashion when the dose reached 160 mg/kg in the healthy mice.

A comparison of the PK parameters of free cefquinome in plasma and thigh, a longer *T*_1/2β_, and lower C_max_ and AUC were found in murine thigh than in plasma under same physical condition and dose. The values of AUC_thigh_ _dialysate_/AUC_plasma_ were 0.76, 0.88, and 0.47 for healthy, neutropenic, and infected mice, respectively. These results indicated that cefquinome had low penetration from the plasma to the thigh muscle when the mice infected by *A. pleuropneumoniae*. There was an obvious influence on the PK of cefquinome in the thigh compared to in plasma under different physical conditions.

## Discussion

In general, free drug pharmacokinetics in the plasma are used to design dosing regimens or correlate efficacy in animal infection models (Griffith et al., 2008; Shan et al., 2014; Guo et al., 2016; Yu et al., 2016; Zhou et al., 2017). Because the plasma collective method is relatively easy and the drugs pharmacokinetics was stable, therefore the pharmacokinetic parameters in plasma are regularly used in pharmacy to design dosage schedules. However, the difference of pharmacokinetics between plasma and tissues is complex, especially in cases of infected organs. Furthermore, only the unbound antimicrobial concentration in tissue can reflect antibacterial efficacy. Therefore, it is necessary and valuable to explore the pharmacokinetics of drugs in tissues. Microdialysis is a microsampling technique that can evaluate free drug concentration in nearly any tissue or species. It is a powerful technology that can facilitate the development of drugs. This technique has been used to explore drug pharmacokinetics in several species, such as pigs (Tyvold et al., 2007; Rottbøll and Friis, 2014; Rottbøll and Friis, 2016; Yang et al., 2017) and rat (Araujo et al., 2008; Bernardi et al., 2017). In the present study, we used microdialysis to determine the pharmacokinetics of cefquinome in the thigh muscle of mice and compared the difference between pharmacokinetics in the plasma and thigh in animal models with different physical statuses.

In our experiment, the *in vitro* recoveries of the probe were not statistically different for different cefquinome concentrations regardless of whether determined by dialysis or retrodialysis. These results indicated that cefquinome did not observably binding with the microdialysis probe. Therefore, the *in vivo* recovery could be determined by retrodialysis (Bernardi et al., 2017).

With regard to *in vivo* recoveries, there was almost no difference among the mouse groups: healthy mice, 39.35%; neutropenic mice, 38.59%; and infected mice, 37.29%. However, these RRs were higher than those associated with *in vitro* recovery (27.52%). This may be explained by the fact that the volume of cells and intercellular space are much larger than Ranger’s solution used *in vitro*. As a result, a low concentration of cefquinome was detected in the thigh dialysate and the RRs were high. The recovery values were stable for 6 h, which indicated that the use of an average value is valid for the determination of the concentrations of cefquinome in the thigh by thigh dialysis.

The plasma protein binding determined by microdialysis was 16.4% in our study. This value was much higher than that reported previously (7.4%) which was determined using ultrafiltration methods (Wang et al., 2014). The difference of plasma protein binding determined by different method has been found by Matzneller et al. (2016). In their study, the values ranged from 75 to 86.8% determined by microdialysis in *in vivo* which was lower than investigated in *in vitro* (>90%) by other researchers. Therefore, in our experiment, we preliminarily considered that the difference of plasma protein binding is probably attributable to the different detection methods and more detailed experiments should be done to explain this phenomenon.

The PK profiles of free cefquinome in plasma were not statistically different among the healthy, neutropenic, and infected mice. This phenomenon was also observed by Liu et al. (2003) who explored the pharmacokinetics of florfenicol in healthy pigs and pigs infected with *A. pleuropneumoniae*. They found that the pharmacokinetic profiles for infected and healthy pigs were not significantly different. This means that the pharmacokinetics of antibacterial drugs in plasma were stable and bacterial infection had little effect on them. Other researchers (Shan et al., 2014; Wang et al., 2014; Guo et al., 2016) reported that the *T*_max_, *T*_1/2β_, *C*_max_, and AUC of cefquinome in mouse plasma were 0.27–0.31 h, 0.31–0.37 h, 28.39–49.5 μg/mL, and 25.26–44.1 μg h/mL, respectively. Our results were similar to these reports.

The pharmacokinetic parameters in thigh were significantly affected by infection. Because of the bacterial infection, the penetration (defined as AUC_thigh_/AUC_plasma_) of cefquinome in infected mice (0.47) from plasma to thigh muscle was much lower than that in the healthy (0.76) and neutropenic (0.88) mice. The main reason for this phenomenon is that bacterial infection can destroy cells and invade into interstitial space and thus less cefquinome permeates into muscular tissue (Kiem and Schentag, 2008). Therefore, it is more rational when design dosages based on the PK of drugs in target organs to treat bacterial infection.

In conclusion, we successfully used microdialysis to determine the pharmacokinetics of cefquinome in the thigh tissues of healthy, neutropenic, and infected mice. The pharmacokinetic profile of cefquinome in the thigh was significantly influenced by the infection. A comparison of the pharmacokinetic parameters in the plasma and thigh muscle between healthy and neutropenic mice suggested that neutrophils may have a negative influence on cefquinome pharmacokinetics in neutropenic mice. In our further study, a mount of studies should be researched to illuminate and promote the application of microdialysis in designing rational dose regimens.

## Author Contributions

HD and LZ conceived and designed the experiments. LZ, LY, and ZH performed the experiments. LZ analyzed the data and drafted the article. XG, XS, and HD contributed to the revision. All authors read and approved the final manuscript.

## Conflict of Interest Statement

The authors declare that the research was conducted in the absence of any commercial or financial relationships that could be construed as a potential conflict of interest.
